# Spatial Expansion of Built-Up Areas in the Beijing–Tianjin–Hebei Urban Agglomeration Based on Nighttime Light Data: 1992–2020

**DOI:** 10.3390/ijerph19073760

**Published:** 2022-03-22

**Authors:** Hua Zhang, Chen Liang, Yuxuan Pan

**Affiliations:** Beijing Key Laboratory of Environmental Remote Sensing and Digital Cities, Faculty of Geographical Science, Beijing Normal University, Beijing 100875, China; cliang_cnu@126.com (C.L.); 202021051044@mail.bnu.edu.cn (Y.P.)

**Keywords:** nighttime light data, spatial expansion, urban area extraction, Beijing–Tianjin–Hebei urban agglomeration

## Abstract

Built-up areas are one of the most intuitive and important indicators used to assess urbanization, the spatial expansion of which is of great significance in depicting the evolution of urban spatial structures. Based on the harmonized Defense Meteorological Satellite Program (DMSP) nighttime light dataset, this paper extracts the spatial distribution of built-up areas and explores the spatial expansion patterns and spatiotemporal evolution regularity of the Beijing–Tianjin–Hebei urban agglomeration from 1992 to 2020. The results show that the spatial comparison method, comparing the extracted area with the government’s statistical area, can accurately determine the optimal threshold of nighttime light and extract urban built-up areas. According to the spatial comparison method, the built-up areas of the Beijing–Tianjin–Hebei urban agglomeration are expanding rapidly from 1992 to 2020, and both expansion speed and expansion intensity have experienced an inverted “U-shaped” growth process. As the core cities of the Beijing–Tianjin–Hebei urban agglomeration, Beijing and Tianjin have been in the later stage of spatial expansion with slower expansion speed but better quality. In contrast, prefecture-level cities and other node cities have rapid expansion speed. The urban space structure of the Beijing–Tianjin–Hebei urban agglomeration has changed from a “monocentric model” to a “polycentric model” to a “metropolitan model”. High-tech industry parks around node cities have become important strongholds of urban space development, leading cities to evolve from monocentric structures to polycentric structures of downtown and industrial parks. The radiation range of core cities expands and spreads to surrounding districts and counties, which inevitably lead to the formation of metropolitan areas.

## 1. Introduction

China has experienced a spectacular and unprecedented process of urbanization since its reform and opening-up process was initiated. The urbanization rate rose from 17.9% in 1978 to 63.9% in 2020, and the urban population increased from 172 million to 902 million. In the process of China’s high-speed urbanization, the rural population, especially the migrant workers, spontaneously converge to cities. A large number of super cities and megacities have emerged, accompanied by negative externalities such as traffic congestion, housing shortage, environmental pollution, accidents, and disasters. The development of small towns and rural areas is relatively lagging behind, and the rural recession such as land abandonment, empty shells, and hollow villages appear frequently. The spatial expansion of built-up areas is not merely an intuitive embodiment of urban expansion but an important representation of the changes in urban system structure and internal spatial structure within a certain period [1]. Furthermore, urban agglomerations are places where the process of urbanization is intensive, and naturally, their expansion possesses indispensable features of complexity and diversity [2]. This paper addresses the spatial expansion of urban agglomerations to highlight the evolution regularity that is critically significant for obtaining an in-depth understanding of the general rules of urban development.

In the existing research, urban spatial expansion analysis typically relies on traditional statistical data and remote sensing data; however, this presents problems, including the statistical complexity, sample selection limitations, and inconsistency in data processing [3,4]. It is notable that urban built-up areas recorded in the China Urban Construction Statistical Yearbook ideally reflect the actual construction and growth of cities, but most of the data are submitted directly to the Ministry of Housing and Urban–Rural Development of China by local statistical departments without following a unified standard. Moreover, the statistical data are numerical, which effectively means the loss of spatial visualization [5]. In recent years, research has proven the feasibility of using remote sensing and GIS techniques in urban sprawl dynamic monitoring. Based on Landsat TM images, a supervised classification method based on support vector machines (SVM) and visual interpretation are applied to depict the spatial pattern of urban expansion from regional to global scales [6,7,8,9,10]. Scholars have represented urban areas with impervious surfaces or construction land when using remote sensing data, which yields insufficient accuracy in urban boundary extraction and difficulty in achieving consistency and comparability in land use data processing. More relevantly, the abovementioned approach defines all construction land equally, but the intensity of socioeconomic activity it carries and its urban vitality vary greatly. Although remote sensing data can reflect the spatial distribution of built-up areas, they have insuperable difficulties in portraying the economic vitality and interactive function of cities.

Nighttime light is a direct reflection of human activity, which enables various lines of inquiry, such as socioeconomic parameter estimation, urban extent monitoring, major event dynamics, and ecological environment assessment. Extracting urban boundaries on the basis of nighttime light observations can precisely identify the active areas of human activity in urban agglomerations and objectively describe both the spatial radiation scope and internal connection strength of urban agglomerations [11,12]. Currently, the most extensively used data resources are the Defense Meteorological Satellite Program/Operational Linescan System (DMSP/OLS) nighttime light for data prior to 2013 and the Visible Infrared Imaging Radiometer Suite (VIIRS) on Suomi National Polar-orbiting Partnership satellite (VIIRS) for data since 2012, published by the National Geophysical Data Center (NGDC) of the National Oceanic and Atmospheric Administration (NOAA). Henderson and Small were among the first geography scholars to use the dynamic threshold of nighttime light in urban extent mapping [13,14]. Since then, a dynamic threshold strategy has become a commonly employed method for analyzing China’s urbanization among domestic scholars. Using DMSP/OLS data, Wang et al. [15] explored the spatial expansion pattern and the driving dynamics of the Beijing–Tianjin–Hebei metropolitan region between 1992 and 2010 and found that its expansion demonstrates a trend of centralization within Beijing, Tianjin, and Tangshan. Li et al. [16] selected the adjusted NPP-VIIRS images as the proxy variable and discussed the circular features and real distribution of the permanent population in Shanghai in more detail. Wang, Li, and Wu [17,18,19] comprehensively analyzed the spatiotemporal pattern and influencing factors of China’s energy consumption, carbon emissions, and air pollution based on the significant correlation between nighttime light data and the intensity of human activity. However, the potential of the historical archive of nighttime light observations has not been fully explored due to the unassailable inconsistency between DMSP/OLS and NPP-VIIRS data in terms of their value and resolution. Integrated and consistent nighttime light datasets have, therefore, rarely been generated in previous studies.

Significant progress has been made in coordinating the development of the Beijing–Tianjin–Hebei region as it rose to the forefront of the national strategy, and scholars pay more attention to the rules of spatial expansion for urban agglomerations in China. The Beijing–Tianjin–Hebei urban agglomeration is not only the largest metropolitan area in northern China but also the growth pole of China’s economy. With the rapid development of urbanization, the influence of cities is increasing, which has led to an enormous amount of research into urban land expansion in the Beijing–Tianjin–Hebei region and its surrounding areas [15]. The existing research can be divided into two parts: first, to enquire into the equilibrium level of regional urban systems based on statistical data; and second, to carry out urban spatial expansion simulation and prediction using remote sensing data. Previously, Sun and Song [20,21,22] characterized the city scale by non-agricultural or urban populations and systematically analyzed the evolutionary features of the city scale structure of the Beijing–Tianjin–Hebei urban agglomeration since the 1980s. The results proved that Beijing and Tianjin constituted the “two poles” of regional development, the monopoly position of large cities was prominent, and the urban system structure presented an “inverted pyramid” shape. In later research, Yang, Chen, and Dong [23,24,25] graphed the distribution of urban land with the aid of high-resolution satellite data, successively verified the polarized expansion process of the Beijing–Tianjin–Hebei urban agglomeration, and pointed out that the distribution of urban areas was dangerously concentrated, with prominent high-order cities and inconspicuous small or medium-sized cities. The leading role of Beijing—the primary city—needs to be strengthened. Xu [26] simulated the future scenarios of urban expansion in Beijing, Tianjin, and Hebei based on the SLEUTH (slope, land use, exclusion, urban extent, transportation, and hillshades) model and found that the urban expansion rate continued to decline, the shrinking trend of cultivated land slowed down, and the difference between inhabited and land areas eased from 2008 to 2020.

The current research focuses on the accurate and objective assessment of the equilibrium of Beijing–Tianjin–Hebei urban expansion and its scale by concentrating on large cities such as Beijing and Tianjin; however, research on the spatial expansion mode and phased characteristics of long-term urban expansion is still lacking. Based on the temporarily extended DMSP nighttime light dataset as well as statistical data of built-up areas and urban populations, this paper determines the optimal threshold of nighttime light using the spatial comparison method by comparing the extracted area with the government’s statistical area and further explores the spatial patterns and dynamic evolution of urbanized areas in the Beijing–Tianjin–Hebei urban agglomeration from 1992 to 2020. By having a stronger appreciation of the stage of urban development and the rationality of urban expansion, the research findings will provide reference information for optimizing regional spatial structure, particularly to comprehensively promote the coordinated and sustainable development of urban agglomerations in China.

## 2. Data and Method

### 2.1. Study Area

**Figure 1:**
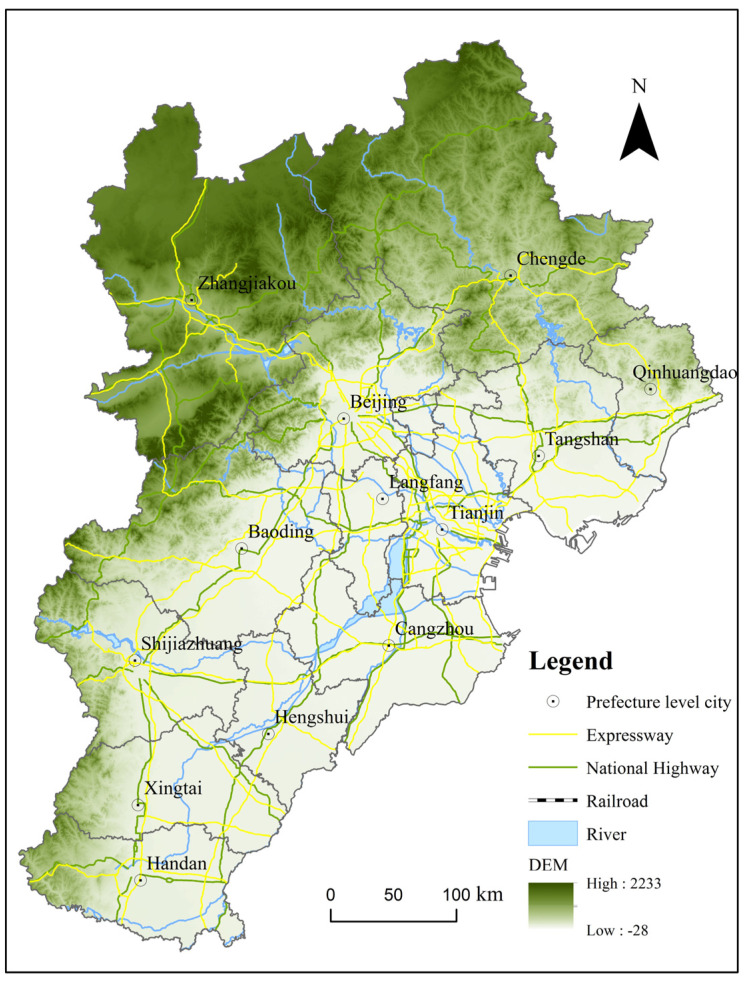
Map of Beijing–Tianjin–Hebei urban agglomeration.

In this study, the Beijing–Tianjin–Hebei urban agglomeration, including 13 cities in all administrative regions of Beijing, Tianjin, and Hebei provinces, was selected as a research area, pursuant to the Coordinated Development Plan of the Beijing–Tianjin–Hebei Urban Agglomeration (Figure 1). Since the reform and opening-up, the Beijing–Tianjin–Hebei urban agglomeration has developed into one of the strongest areas in terms of comprehensive strength and is simultaneously an important force leading China’s socioeconomic development and urban construction due to its superior geographical location and the policy support it has received. As of 2020, it represents 7.81% of the population and 2.25% of the landmass and accounts for nearly 8.46% of the national economy.

Since 1978, regional and urban–rural disparities in the Beijing–Tianjin–Hebei urban agglomeration enticed an unprecedented wave of people to migrate from the countryside to urban areas to pursue a more prosperous lifestyle [27]. The urbanization rate rose from 20.91% in 1978 to 68.61% in 2020, representing an annual increase of 1.14 percentage points; the total urban population grew from 13.90 million in 1978 to 75.73 million in 2020, an average annual increase of 10.59%. In addition, the urban built-up areas experienced exponential and remarkable growth, expanding from 1389 km^2^ in 1993 to 4228 km^2^ in 2019, an increase of 2.04 times [28].

### 2.2. Data Source

The stable light and radiance calibrated products from DMSP/OLS and NPP-VIIRS are primary data sources in urban expansion mapping [12,29]. However, we should bear in mind that there is a vast difference in attributes between DMSP/OLS and NPP-VIIRS nighttime light images. First, compared with DMSP/OLS data, both spatiotemporal and radiometric resolutions of NPP/VIIRS data are notably improved. Second, DMSP/OLS images are jointly captured by six generations of satellites where digital number (DN) values fluctuate abnormally in different years, which leads to overglow effects in central urban areas. Third, whereas NPP-VIIRS provides spatially more explicit lights within the city, the low-value detection capability of the images may result in accidental noise. In summary, a harmonized nighttime light dataset with convincing comparability can provide rudimentary support for accurately extracting the urban spatial scope.

The nighttime light dataset employed in this research comes from a study by Li et al. [16], who generated an integrated and consistent global nighttime light dataset by harmonizing the intercalibrated nighttime light and observations from DMSP/OLS data and the simulated DMSP/OLS-like nighttime light observations from NPP-VIIRS data. The temporal span of the dataset is from 1992 to 2020, while its spatial resolution is 30 arc-seconds, with DN values ranging from 0 to 63. Excluding noise from the aurora, fires, boasts, and other temporal lights, the extended DMSP/OLS dataset shows high sensitivity to the low-intensity lights emitted by urban lights, small-scale residential areas, and traffic flow, which makes it obviously different from a dark, rural background. Research has confirmed that harmonized DMSP/OLS nighttime light data could be used to support studies monitoring the long-term dynamics of human activity, such as urban expansion, carbon emissions, electricity consumption, and light pollution, at local to global scales [16].

Moreover, basic geographic and urban statistical data are included in this study. Basic geographic data at a 1:1,000,000 scale derived from the Resource and Environment Science and Data Center of Institute of Geographic and Natural Resources Research (https://www.resdc.cn/, accessed on 5 February 2022) are applied to extract the vector boundary of the Beijing–Tianjin–Hebei urban agglomeration. The built-up areas of municipal districts at the prefectural level collected from the China Urban Construction Statistical Yearbook in 1993, 2000, 2010, and 2019 are used for the delimitation of urban spatial scope and further analysis of urban expansion trends.

### 2.3. Built-Up Areas Extraction

The government’s statistical department releases data relating to urban built-up areas every year—built-up areas here refer to a large developed and constructed area that features municipal public facilities. Previous studies indicated that the extent of urban areas extracted by the spatial comparison method by comparing the extracted area with the government’s statistical data was highly consistent with the relatively concentrated areas of urban land identified by the municipal government [4]. In this research, it is implied that the threshold of nighttime light needs to be dynamically adjusted according to the distribution of urban built-up areas, which means we need to continuously reduce the threshold of nighttime light from 63 to attain the spatial distribution of built-up areas of different cities in different years.

Following this approach, we minimize the error between the extraction results and the statistical scope to determine the optimal threshold for effectively and efficiently extracting urban areas in a specific year. Taking Beijing as an example, we can see from the latest statistical data released by the government that the built-up areas of Beijing reached 1469 km^2^ in 2020. Referring to this data, we continuously adjusted the threshold of nighttime light within the urban extent of Beijing until it was reduced to 61; the number of built-up areas is the closest to statistical data, reaching 1642.41 km^2^. Therefore, we determined 61 as the optional threshold of nighttime light for extracting the built-up areas of Beijing in 2020. At this time, the extraction proportion of built-up areas reached 111.08%, according to the statistical data, and the spatial distribution of which is shown in Figure 1. Therefore, we compare the built-up areas published by the statistical department above the prefecture level with the urban scope extracted through a specific threshold and ultimately obtain the spatial pattern of urban areas in the Beijing–Tianjin–Hebei urban agglomeration in 1992, 2000, 2010, and 2020, which we consider to be one of the most distinctive aspects of this research.

### 2.4. Urban Expansion Indices

Expansion speed is the annual growth rate of urban built-up areas during a particular study period, which identifies the absolute disparities in urban expansion between different cities during the study period [15].

(1) $$V\;=\frac{UL_{i\;+\;t}-UL_{i}}{t}$$

where *UL_i_* and *UL_i + t_* are urban areas in years *i* and *i + t*, respectively, and *t* represents the interval of the study years.

Expansion intensity is the proportion of the urban built-up areas in the total area over a period, which represents the relative disparities in urban expansion between different cities during the study period [15].

(2) $$\mathrm{EI}\;=\frac{UL_{i\;+\;t}-UL_{i}}{S\times t}\times 100$$

where *UL_i_* and *UL_i + t_* are urban areas in the years of *i* and *i + t*, respectively; *S* is the total area; and *t* represents the interval of study years. The higher the EI value is, the stronger the urban land expansion will be.

Compactness is widely used to quantitatively evaluate the spatial conformation of a city. It can reveal the degree of spatial concentration of patches in built-up areas [8].

(3) $$\mathrm{BCI}\;=2\sqrt{\pi A_{i}}/P_{i}$$

where *A_i_* is the area of city *i* and *P_i_* is the perimeter. The BCI value falls in the range of 0–1; a higher value demonstrates higher compactness of a city, while a lower value indicates a looser urban pattern. In general, when the city is expanding rapidly, the compactness will decrease; however, as the city begins to fill internally, the compactness will increase.

The fractal dimension value is an indicator that reflects the regularity of city boundaries. It can assist in distinguishing three developing stages of urban expansion: external expansion, internal filling and stable development [8].

(4) $$S\;=2\ln\left(\frac{P_{i}}{4}\right)/\ln\left(A_{i}\right)$$

where *A_i_* is the area of city *i* and *P_i_* is the perimeter. If the S value is less than 1.5, the city boundary tends to be smooth. If the S value is more than 1.5, the city boundary is more indented. When the value becomes closer to 1.5, the city is in a state corresponding to Brownian random motion, and the closer it is to this level, the more complicated and unstable the urban morphology will be.

## 3. Built-Up Areas Extraction and Spatial Expansion Features

### 3.1. Results of Built-Up Areas Extraction

Urban expansion analysis shows that from 1992 to 2020, the spatial scope of built-up areas in the Beijing–Tianjin–Hebei urban agglomeration expanded notably, and the number of patches increased significantly, nearly doubling from 53 in 1992 to 105 in 2020. The identified patches of built-up areas are mainly distributed in the municipal districts of cities at the prefecture level or above, and some suburban transitional zones or county seats with intensive economic activity are also recognized as built-up areas. However, due to the limitations of administrative definition, although a county has a developed economy and intensive human activities, it cannot be determined as a city. This is why nighttime light data (like DMSP/OLS data) can more truly reflect human activities and their impact on the natural environment as well as human societies than remote sensing data.

**Figure 2:**
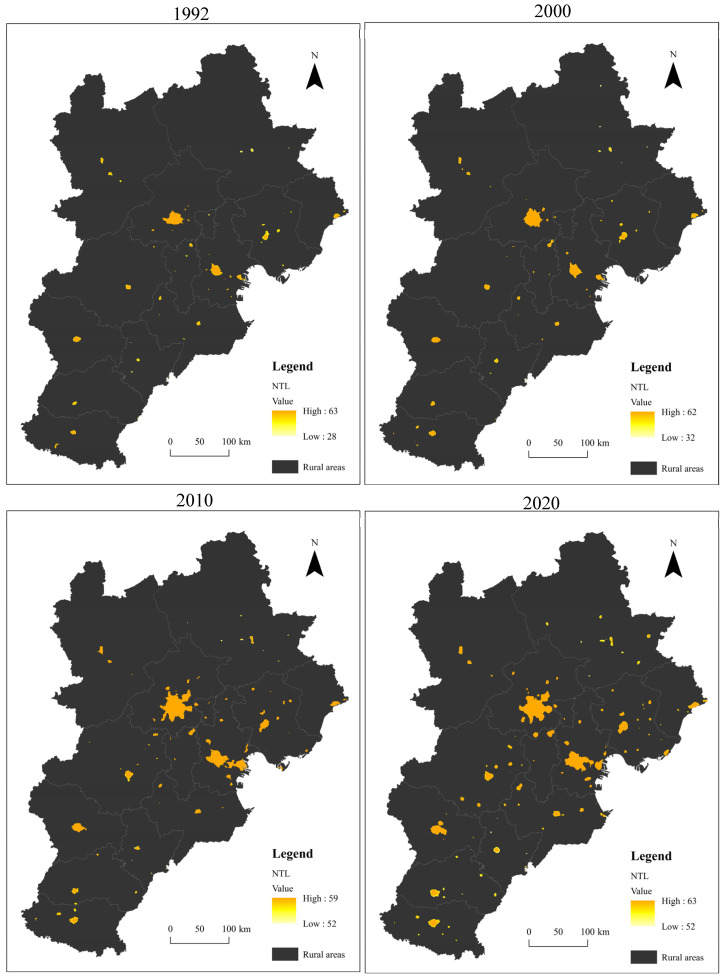
Urban Built-up Areas Extraction Results in the Beijing–Tianjin–Hebei Urban Agglomeration in 1992, 2000, 2010 and 2020.

Fortunately, the extraction results are tightly coupled with the statistical data published by the government. The extraction proportion of built-up areas within municipal districts reached 108.51%, 124.52%, 119.96% and 106.25% in 1992, 2000, 2010 and 2020, respectively (Figure 2).

According to the spatial distribution of built-up areas, the two megacities—Beijing and Tianjin—are highly clustered and have become the core of the Beijing–Tianjin–Hebei urban agglomeration, followed by Tangshan, Shijiazhuang, and Baoding. In addition, Xingtai, Handan, and Cangzhou expanded rapidly in the later stage.

### 3.2. Rapid Expansion of Built-Up Areas in the Beijing–Tianjin–Hebei Urban Agglomeration

The extent of the Beijing–Tianjin–Hebei urban agglomeration has expanded significantly, and the intensity of its economic activity has increased over the past 30 years. The remarkable expansibility of its urban area is reflected in the tremendous increase in built-up areas, which have been 1577.30 km^2^ in 1992, 2047.06 km^2^ in 2000, 4381.15 km^2^ in 2010, and 5418.69 km^2^ in 2020. The corresponding DN values are 127,837, 171,980, 333,125 and 476,995, respectively, a 273.13% jump from 1992 to 2020. The average nighttime light intensity rose from 81.05 to 88.03, thus indicating an increase in the strength of economic activity. Since the 1990s, China’s market-oriented economy has developed vigorously and entered a rapid development phase. Given its resource endowments, locational advantages, and policy support, the Beijing–Tianjin–Hebei urban agglomeration includes a large number of high-quality industries, talent, and capital elements and is becoming one of the most prominent areas of urban spatial expansion in China, the visible sign of which is constantly expanding urban area and booming economic endeavors [25,30,31].

This study claims that both the expansion speed and intensity of the Beijing–Tianjin–Hebei urban agglomeration increased first and then decreased—as such, they experienced a gradual and inverted “U-shaped” growth process (Table 1). China was in transition from 1992 to 2000, when it began to move away from a planned economy towards a market economy, and thus, the driving force of urbanization had not been fully stimulated. The expansion speed in this period was only 58.72 km^2^/a (square kilometers per year), the expansion intensity was less than 0.03, and the expansion of built-up areas remained sluggish. From 2000 to 2010, the Beijing–Tianjin–Hebei area entered a new stage that boosted economic growth and accelerated urbanization by developing a manufacturing industry accompanied by a great increase in industrial land. In the so-called “aggressive” growth stage of its urban area, the expansion speed rose to 233.41 km^2^/a, and the expansion intensity showed a swift increase to 0.11 [32]. After 2010, China strengthened the regulation and control over urban expansion by focusing on controlling the scale of newly built-up areas in the Beijing–Tianjin–Hebei area. Today, the urban expansion rate of the region has dropped to 103.75 km^2^/a, while the expansion intensity is merely 0.05.

### 3.3. Great Disparities in the Spatial Expansion of Prefecture-Level Cities

#### 3.3.1. Differences in the Spatial Scale of Cities

Beijing and Tianjin, the two core cities, occupy more than 50% of the built-up areas and the corresponding DN value of the whole region—most notably, the expansion speed far exceeds the pace of other cities, and the proportion is still rising. However, Beijing and Tianjin have always been the top two cities in terms of urban expansion and DN value in the Beijing–Tianjin–Hebei area. The urban areas of Beijing and Tianjin in 1992 were 498.46 km^2^ and 351.44 km^2^, accounting for 31.60% and 22.28% of the total, respectively, and the DN values reached 44,399 and 29,723 and accounted for 34.73% and 23.25% of the total, respectively. In 2020, the built-up areas of the two cities increased to 1642.41 km^2^ and 1164.95 km^2^, with the proportions growing to 30.31% and 21.50%, respectively, while the DN values were 44,399 and 29,723, accounting for 34.73% and 23.25%, respectively. It can be seen that the urban areas in Beijing and Tianjin have tripled in size over the past 28 years, their expansion speed reached 40.86 km^2^/a and 29.05 km^2^/a, respectively, and their expansion intensity is greater than 0.25.

However, Tangshan, Shijiazhuang, and Baoding are in the second echelon of the spatial expansion of built-up areas in the Beijing–Tianjin–Hebei urban agglomeration—surpassed only by Beijing and Tianjin—and their urban area and corresponding DN values greatly exceeded one-fifth of the total area. More specifically, Tangshan, Shijiazhuang, and Baoding ranked third, fourth, and eighth, respectively, in terms of their urban scale in 1992, the built-up areas of which reached 149.82 km^2^, 95.91 km^2^, and 61.61 km^2^, accounting for 9.50%, 6.08% and 3.91% of the total, respectively. Furthermore, the DN values were 10,697, 7983, and 4850, with proportions of between 3 and 10%. It is worth noting that Baoding jumped to fifth place by the end of 2020 and was slightly lower than Tangshan and Shijiazhuang. The urban areas of the respective cities increased rapidly in the previous 30 years and reached 447.36 km^2^, 364.75 km^2^, and 336.74 km^2^, accounting for 8.26%, 6.73%, and 6.21% of the total, respectively, while the DN values grew to 39,306, 32,372 and 29,299, respectively, accounting for over 8% of the total.

Located at the north and south ends of the region, Hengshui and Zhangjiakou have faced the dilemma that their steady and uninterrupted socioeconomic development is highly restrained by limited space, as they have the lowest built-up areas and DN values among the 13 cities. In 1992, the built-up areas of Hengshui and Zhangjiakou occupied only 74.91 km^2^ and 30.80 km^2^, the proportions of which were 4.75% and 1.95%, respectively, and the DN values were only 5318 and 1850, accounting for well below 5% and 2% of the total, respectively. In 2020, the urban area of the two cities expanded slightly, reaching 119.72 km^2^ and 96.61 km^2^, accounting for 1.78% and 2.21% of the total area of Beijing, Tianjin, and Hebei, respectively. Despite the impressive growth of their DN values to 7937 and 10,555, the expansion speed of Hengshui and Zhangjiakou was never comparable to that of the whole region, as the proportion was still less than 3%. Notably, the built-up areas in other cities are within 150 to 300 km^2^; concurrently, their DN values range from 13,000 to 25,000.

#### 3.3.2. Great Disparities of Expansion Speed

The exponentially expanding area concentrated in Beijing, Tianjin, Tangshan, Baoding, and Shijiazhuang and the spatial expansion of the megacities are particularly prominent in the metropolitan area. From 1992 to 2020, these cities ranked among the top five with scales of 1143.94 km^2^, 813.50 km^2^, 297.54 km^2^, 275.13 km^2^ and 268.83 km^2^, while the DN values reached 102,439, 74,385, 28,609, 24,449 and 24,389, respectively. In contrast, Zhangjiakou has the slowest urban scale growth with a 44.81 km^2^ built-up area, and the growth of the DN value is only 5237, which is even less than the 6% of Beijing. The expansion speed of urban land in Zhangjiakou is barely 1.60 km^2^/a, and the expansion intensity is lower than 0.01.

#### 3.3.3. Periodic Differences of Urban Expansion

This paper takes 2010 as a transition point, after which the area developed much more rapidly. The findings show that the rapidly expanding area in the Beijing–Tianjin–Hebei urban agglomeration was located in the two megacities—Beijing and Tianjin—before 2010; thereafter, the main area gradually transferred to node cities, such as Cangzhou, Shijiazhuang, Baoding, and Xingtai (Table 2). From 2000 to 2010, the expansion speeds of Beijing and Tianjin increased significantly and reached 94.72 km^2^/a and 72.46 km^2^/a, respectively; their expansion intensity reached 0.60. The highest expansion speeds from 2010 to 2020 appeared in Cangzhou, Shijiazhuang, and Baoding, with average annual expansion areas of 18.55 km^2^, 13.86 km^2^, and 13.58 km^2^, respectively; the expansion intensities of Cangzhou, Shijiazhuang, and Xingtai reached peaks of 0.13, 0.09 and 0.09, respectively. The comparative analysis of the periodic differences in the expansion of prefecture-level cities reveals that the expansion speeds and intensities of Beijing, Tianjin, and Tangshan increase first and then decrease; however, the expansion of Langfang continues to slow or even reverse with the accelerated expansion of other cities. Accordingly, we find that the spatial distribution of urban areas in Beijing and Tianjin intensified coincident with the pace of urbanization in core cities due to their drastic radiation effect. When other cities enter a period of accelerating urbanization, the northwest mountain area unexpectedly falls into the trap of mediocre growth; that is, the unique landscape characteristics may put forth a higher requirement for urban planning and construction over the long run.

From the variation in nighttime light, Beijing and Tianjin become the fastest growing areas, where the DN values reached 77,360 and 59,312 from 2000 to 2010, respectively, accounting for nearly 70% of the total DN value of the Beijing–Tianjin–Hebei urban agglomeration. In addition, increases in DN values per unit area of 81.67/km^2^ and 81.86/km^2^ were obtained for these two cities. The growth trend of Beijing and Tianjin for the next 10 years remained at the forefront, with increments of 15,651 and 6912, respectively, and the DN value per unit increased to 1315.04/km^2^ and 394.92/km^2^. Therefore, we compare the spatial expansion of built-up areas in the same period and find that the expansion mode of core cities validates an approach favoring limited speed but improved quality; nonetheless, that of node cities is characterized by denotative expansion.

## 4. Evolutionary Characteristics of the Spatial Structure of the Built-Up Areas

### 4.1. 1992–2000: Monocentric Expansion

**Figure 3:**
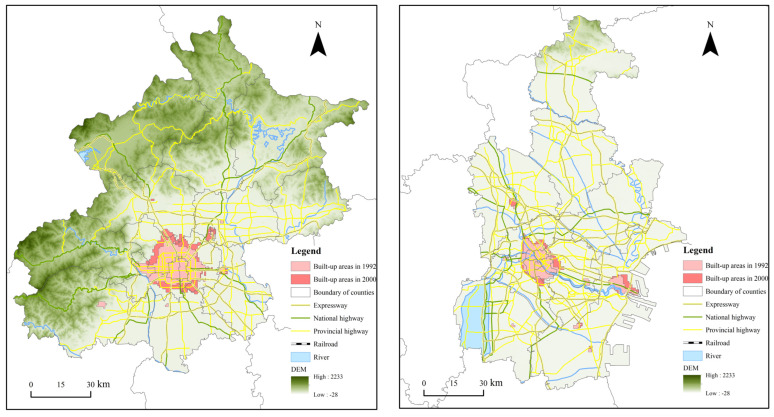
Spatial Expansion Patterns of Beijing and Tianjin from 1992 to 2000.

The expanding built-up areas in the Beijing–Tianjin–Hebei urban agglomeration at the initial stage of urbanization were predominantly located in municipal districts at the prefectural level or concentrated in the counties at the periphery of Beijing, Tianjin, Tangshan, and Shijiazhuang. The number of those patches increased from 53 in 1992 to 54 in 2000 without significant change; however, we should emphasize that the spatial distribution of built-up areas presented a fairly high level of centralization (Figure 3). In the early 1990s, construction in the Beijing–Tianjin–Hebei region was relatively slow, and the built-up areas were both scattered and small in scale. They were mainly located in the central urban areas and expanded along the traffic artery. Relying on the construction of old and heavy industrial bases, the central areas of Beijing (Dongcheng, Xicheng, Haidian, Chaoyang, Shijingshan, and Fengtai districts), Tianjin (Heping, Hedong, Hexi, Nankai, Hebei, and Hongqiao districts), Shijiazhuang (Qiaoxi, Xinhua, Changan and Yuhua districts) and Tangshan (Lubei, Lunan and Kaiping districts) are connected and form a unified single-center region with a consistent socioeconomic development trend. The urban fringe area, including Shunyi, Wuqing, Binhai, Fengrun, Zunhua, Yutian, and Zhengding, has gradually grown in size and become a major focus of the utilization of urban space in the Beijing–Tianjin–Hebei urban agglomeration.

The calculation results of urban expansion indices demonstrated that the compactness of Beijing, Tianjin, Qinhuangdao, Xingtai, Zhangjiakou, Cangzhou, and Hengshui increased, and the fractal dimension decreased, while the spatial pattern was increasingly simplified. The well-organized urban space was constantly filled, connected, and compact. In contrast, the compactness of Tangshan, Shijiazhuang, Handan, Baoding, Chengde, and Langfang decreased, and the fractal dimension increased, which led to a dynamic and complex tendency in the evolution of the distribution of the urban area. The single center with an expanding circle still extended along the traffic artery. Overall, the spatial expansion pattern of built-up areas in Beijing, Tianjin, and Hebei was comparatively orderly, and an urban agglomeration with an appropriate scale has been formed since the 1990s.

### 4.2. 2000–2010: Polycentric Expansion in Core Cities and Monocentric Expansion in Node Cities

**Figure 4:**
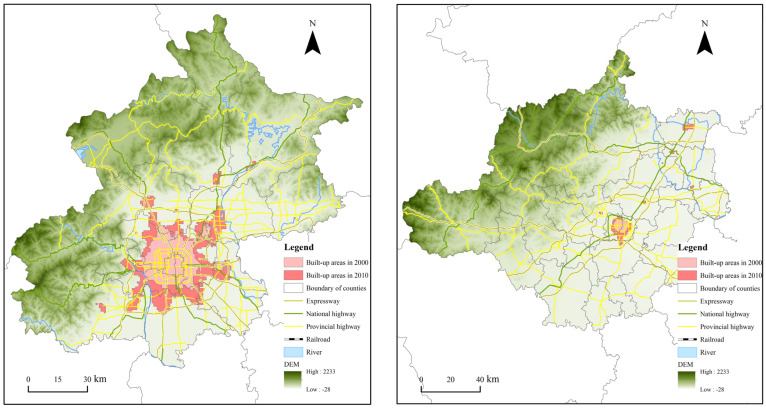
Spatial Expansion Patterns of Beijing and Baoding from 2000 to 2010.

As the core cities adjusted their spatial organization structure from a “monocentric model” to a “polycentric model” from 2000 to 2010, the built-up areas in regional central cities such as Langfang, Baoding, Shijiazhuang, Qinhuangdao, Shijiazhuang, and Chengde were distributed discontinuously as spots or lines owing to the radiation effect of core cities. The patches of urban areas were booming in this period and nearly doubled from 54 to 83, which verifies the trend of decentralization in the current process of urban growth (Figure 4). The counties at the periphery of Beijing, Tianjin, Tangshan, and Shijiazhuang were the main carriers of urban spatial expansion in the region. Meanwhile, the embryonic areas of Fangshan, Huairou, and Pinggu in Beijing, Baodi and Jizhou in Tianjin, Qianxi, Lunan and Qianan in Tangshan, Luancheng and Xinji in Shijiazhuang appeared one after another. With the constant improvement of the traffic network layout, the population mobility between Langfang, Baoding, Beijing, and Tianjin increased, and the economic interconnectedness was improved. The spatial distribution of urban built-up areas showed a linear series trend covering the central area of Baoding (Jingxiu, Lianchi, Mancheng, Qingyuan, and Xushui), Langfang (Guangyang and Ancheng), and other node counties, such as Dingxing, Gaobeidian, Zhuozhou, Sanhe, Xianghe, and Bazhou. Accordingly, the widened radiation scope of Beijing and Tianjin spread to the fringe area, which led to the formation of metropolitan areas, and the characteristics of regional integration development began to appear.

From the change of compactness and fractal dimension, the compactness of all cities other than Zhangjiakou decreased and the fractal dimension increased, which suggests that urban expansion was in a disordered state with the built-up areas expanding along traffic arteries and that the complexity of space conformation and the tortuosity of city boundaries rose sharply in the beginning of 21th century.

### 4.3. 2010–2020: Metropolitan Areas Emerging in Core Cities and Polycentric Expansion in Node Cities

**Figure 5:**
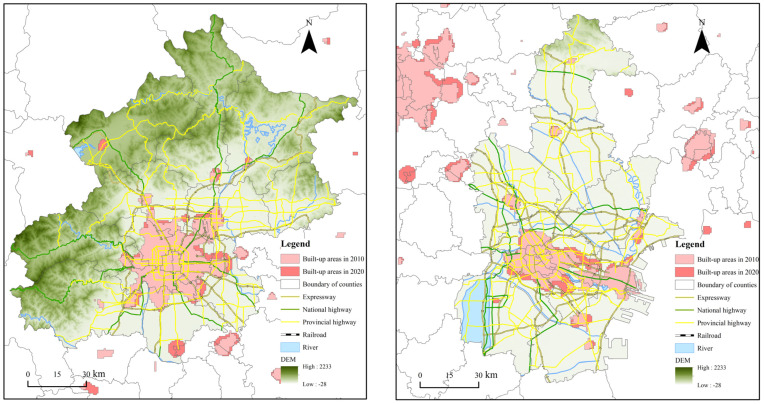
Spatial Expansion Pattern of Beijing and Tianjin from 2010 to 2020.

Infill growth always occurred in the core cities of the Beijing–Tianjin–Hebei urban agglomeration throughout the study period; however, the constantly emerging “new towns” and “satellite cities” at the periphery of node cities ended up becoming the main carriers of urban spatial expansion in the region. With the growing trend of decentralization, the patches of urban areas rose by almost a quarter, reaching 105 by the end of 2020 (Figure 5). The expansion of Beijing and Tianjin decelerated remarkably, and the surrounding city-and-town concentrated area extended to three northern counties and the central urban area of Langfang, together with Dingxing, Gaobeidian, and Zhuozhou in Baoding, which is an ideal foundation for forming a core-periphery urban spatial structure. In the new areas in Tianjin and Binhai, there were urban areas connecting Caofeidian in Tangshan and Huanghua in Cangzhou, thus affirming the state of the metropolitan area with Tianjin as its core. The built-up areas of Baoding, Shijiazhuang, Handan, Tangshan, Qinhuangdao, and other node cities in front of the Yanshan and Taihang Mountains extended to the suburbs with the central urban areas as their core, which has fuelled the transition of the spatial organization from a monocentric to a polycentric mode—a symptom of periodic differences in urban expansion.

The Outline of the Beijing–Tianjin–Hebei Collaborative Development Plan issued in 2015 requires all cities to deepen their industrial structure—as core cities, Beijing and Tianjin improve through industrial upgrading, and the surrounding cities undertake industrial transfer to realize denotative expansion. The construction of integrated transport hubs and industrial parks has not only provided a platform for the formation of high-tech industry development belts along rivers and ports but has also become an important aspect in developing and utilizing urban space. Furthermore, the Planning of Coordinated Development of Tongzhou District in Beijing and Sanhe, Dachang, and Xianghe in Hebei notes that the Yanjiao group will take advantage of radiation and the driving effect of Tongzhou district on the surrounding areas to reasonably undertake Beijing’s noncapital function. In 2020, the built-up area of the Yanjiao Group was 29.40 km^2^, and that of the new areas in Xiongan and Chongli for the 2022 Winter Olympics was 32.90 km^2^ and 37.10 km^2^, respectively. Consequently, the frameworks of “One Body with Two Wings”, including the new area in Xiongan and the sub-CBD in Beijing, and the “Two Wings of Hebei”, which contains the new area in Xiongan and the Chongli area in Zhangjiakou, have been effectively completed [33,34].

In our analysis of compactness and fractal dimensionality, we find that core cities have been in the infill stage of growth as their compactness increased and their fractal dimensionality decreased. This is consistent with the policy on the reconstruction of villages in cities or shanty towns in the Eleventh Five-Year Plan and the Twelfth Five-Year Plan, which verifies a moderating trend of disorderly expansion of the cities. Nevertheless, in Shijiazhuang, Qinhuangdao, Handan, Xingtai, Baoding, Chengde, Cangzhou, and other node cities, the compactness remains unchanged or decreases while the fractal dimensionality significantly increases, which proves that the booming high-tech industry parks around the central areas have improved the changeability and complexity of the urban spatial form. In turn, the spatial expansion pattern of the multifunctional cluster in the Beijing–Tianjin–Hebei urban agglomeration keeps increasing in size.

## 5. Conclusions

Based on the harmonized DMSP-like nighttime light dataset, this paper explores the spatiotemporal expansion patterns of the Beijing–Tianjin–Hebei urban agglomeration in 1992, 2000, 2010, and 2020. In addition, we further explore the spatial expansion mode and structural properties of urban areas and draw the following conclusions:

(1) The spatial expansion of the Beijing–Tianjin–Hebei urban agglomeration has strong momentum. Specifically, both expansion speed and expansion intensity first increased and then decreased over the study period. Beijing and Tianjin, the two core cities, occupy more than 50% of the built-up areas and the corresponding DN value of the whole region. The rapidly expanding cities in the Beijing–Tianjin–Hebei region are Tangshan, Baoding, and Shijiazhuang, while Hengshui and Zhangjiakou, located at the north and south ends, have failed to gain momentum. From the above analysis, we found that there is a profound relationship between urban spatial expansion and economic growth. From 1992 to 2000, China was against a background of slowing growth—moving away from a planned economy towards a market economy—and the driving force of urbanization had not been fully stimulated yet. However, the Beijing–Tianjin–Hebei area entered a new stage during the following decade that boosted economic growth and accelerated urbanization progress by developing its manufacturing industry. In 2014, the coordinated development of Beijing, Tianjin, and Hebei rose to the forefront of the national strategy. The three areas established multilevel cooperation since the implementation of the policy to remove Beijing’s noncapital functions through an industrial connection and the synchronous construction of educational and medical resources in the central cities of Beijing, Tianjin, Langfang, Baoding, and Tangshan.
(2) The spatial expansion of built-up areas in the Beijing–Tianjin–Hebei urban agglomeration demonstrated an obvious periodic characteristic, which means that the expansion of core cities such as Beijing and Tianjin is performed with slower speed but better quality, while that of Cangzhou, Shijiazhuang, Baoding, Xingtai and other node cities are characterized by rapid expansion speed. The most rapidly expanding area was located in two megacities—Beijing and Tianjin—from 1992 to 2010, when the expansion speed reached 94.72 km^2^/a and 72.46 km^2^/a, respectively, and the expansion intensity reached 0.60, thus demonstrating a tendency of denotative expansion. After 2010, the expansion speed and intensity showed a downwards trend; however, the corresponding DN value increased steadily, so we found that the expansion mode of core cities followed connotative promotion during this period, while that of node cities was characterized by denotative expansion with high speed but inferior quality.
(3) The spatial organization of the Beijing–Tianjin–Hebei region follows the evolution law of the monocentric model-polycentric model-metropolitan model. In the initial stages of urbanization, the expanding built-up areas were predominantly located in the municipal districts at the prefectural level or concentrated in the counties at the periphery of Beijing, Tianjin, Tangshan, and Shijiazhuang. In the 1990s, the strengthening of administrative barriers led to a free-for-all in urban governance, a lack of connection between regions, and insufficient vitality in economic development. The spatial organization structure, particularly in core cities, is presented as a single cluster with circling expansion. As cities adjust their spatial organization structure from a “monocentric model” to a “polycentric model”, the built-up areas in regional central cities such as Langfang, Baoding, Shijiazhuang, Qinhuangdao, Shijiazhuang, and Chengde are distributed discontinuously as spots or lines owing to the radiation effect of core cities. The urban expansion was in a disordered state from 2000 to 2010, with a single cluster expanding along the traffic artery. Infill growth always occurred in the core cities after 2010; however, the constantly emerging “new towns” and “satellite cities” around node cities ended up becoming the main carriers of urban spatial expansion in the region. The spatial organization of metropolitan areas appears gradually over time.

Based on the harmonized DMSP-like nighttime light datasets, this paper determines the optimal threshold of nighttime light using the spatial comparison method, which can optimally reflect the economic dynamism and spatial heterogeneity of urban areas. This laid an indispensable foundation for grasping the spatial evolution of urban agglomerations and realizing the healthy and sustainable development of the region [35]. There is an upper limit on the value of DMSP / OLS nighttime light data due to the limitation of DN value. As a result, the statistical DN values in Beijing and Tianjin may be lower than their actual values. However, the total amount of patches in urban built-up areas will continue to increase with the continuous expansion of the urban extent. Even if the intensity of nighttime light in the inner city is saturated, both DN values and built-up areas in Beijing and Tianjin are still far higher than those in other cities. Therefore, the uncertainty caused by the booming effect will not change the existing conclusions, while DMSP/OLS data still have unique advantages in characterizing the differences of built-up area expansion between different cities.

Priorities for future research include the extraction techniques of urban built-up area information to find an accurate, simple, and efficient method for large-scale urban boundary identification, which is the crux of exploratory spatial expansion analysis by employing a multisource nighttime light dataset. In this research, we discuss the spatial expansion pattern of the Beijing–Tianjin–Hebei urban agglomeration in multiple respects. Nevertheless, the formation mechanism of expansion differences in combination with the actual situation of various regions has not been involved. The discussion on the driving force of urban expansion should be enriched as the first prerequisite to meet the needs of “intensive expansion” and “smart growth”.

## Figures and Tables

**Table 1:** Changes in City-scale Expansion of the Beijing–Tianjin–Hebei Urban Agglomeration from 1992 to 2020.

Period	Increment of Built-Up Areas (km^2^)	Increment of DN Value	Expansion Speed (km^2^/a)	Expansion Intensity
1992–2000	469.76	111,251	58.72	0.03
2000–2010	2334.10	193,764	233.41	0.11
2010–2020	1037.53	44,143	103.75	0.05

**Table 2:** Differences in Urban Expansion in the Beijing–Tianjin–Hebei Urban Agglomeration from 1992 to 2020.

City	Expansion Speed km^2^/a			Expansion Intensity			Increment of DN Value /km^2^		
	1992–2000	2000–2010	2010–2020	1992–2000	2000–2010	2010–2020	1992–2000	2000–2010	2010–2020
Beijing	23.10	94.72	1.19	0.14	0.58	0.01	51.01	81.67	1315.04
Tianjin	8.93	72.46	1.75	0.08	0.62	0.01	114.29	81.86	394.92
Shijiazhuang	2.89	10.71	13.86	0.02	0.07	0.09	62.86	12.89	2.41
Tangshan	3.94	16.59	10.01	0.03	0.12	0.07	441.29	139.09	25.36
Qinhuangdao	0.96	2.38	5.32	0.01	0.03	0.07	157.66	13.28	7.40
Handan	4.11	6.09	6.51	0.03	0.05	0.05	821.06	199.97	53.90
Xingtai	1.58	6.79	11.41	0.01	0.05	0.09	301.33	89.43	21.16
Baoding	1.93	12.39	13.58	0.01	0.06	0.06	1020.74	146.64	14.27
Zhangjiakou	0.96	1.61	2.10	0.00	0.00	0.01	148.29	13.66	9.28
Chengde	2.80	2.52	11.06	0.01	0.01	0.03	1208.29	194.51	71.32
Cangzhou	1.23	1.96	18.55	0.01	0.01	0.13	733.30	66.74	11.92
Langfang	4.99	4.55	3.50	0.08	0.07	0.05	391.07	198.04	20.92
Hengshui	1.31	0.63	4.90	0.01	0.01	0.06	103.42	19.80	30.25

## Data Availability

The nighttime light dataset employed in this study are openly available in FigShare at https://doi.org/10.6084/m9.figshare.9828827.v2 [16], accessed on 5 February 2022.
